# Book Reviews

**Published:** 1904-05

**Authors:** 


Social Diseases and Marriage. Social Prophylaxis. By Prince A. Morrow, M. D., Emeritus Professor of Genitourinary Diseases in the University and Bellevue Hospital Medical College, New York; Surgeon to the City Hospital. Octavo, pp. 390. Lea Brothers & Co., New York and Philadelphia. 1904.

The subject dealt with by Morrow in this book has been attempted often before, sometimes extensively and again scantily, but never more delicately, scientifically, or thoroughly; moreover, it is presented in a way that makes it intelligent reading for educated men and women outside of the medical profession. This latter point is important, because any reform of statute or opinion regarding the propagation of social diseases must have the support of public opinion formed upon an intelligent understanding of the question in all its bearings in relation to community.

The dangers that venereal diseases introduce into the marriage state have received considerable attention of late, and they are becoming better and better understood by physicians; but, alas! for the laity, how little they appreciate the risks such diseases entail. Few young men are aware that gonorrhea is one of the most dangerous infections of its class, and that its results if neglected, or uncured, if it becomes chronic, are more lasting than any other venereal disease. If the young wife becomes infected by a gleety husband and her tubes take on suppurative disease, nothing short of excision, in many instances, will effect a cure. Sterility and woe are penalties often inflicted on an innocent woman for the sins of her lecherous husband. Who shall say the state has not some duty to perform looking toward the prevention of this direful state of affairs?

The dangers, however, are not alone to the wife, but the offspring suffer as well, if the infection be syphilitic; hence, if the sins of the father are to be prevented from being visited upon his children, some safeguard must be thrown around the marriage contract. It is a most difficult problem to deal with and possibly it never will be fully solved. The Journal is inclined to agree with Simeon Ford, who asserts that, "the puritanical spirit is as yet too strong within us to permit vice to be legalised and regulated and kept within bounds. We'd rather go on being hypocrites and let blackmailers flourish and dishonest folk fatten."

In spite of all, however, the medical profession has a duty to perform which is well stated by Morrow to have for its object the protection of the innocent and helpless from venereal disease, and to safeguard marriage from its dangers, affirming that in the fulfilment of this duty the highest ideals of preventive medicine are realised. The presentation of the subject scarcely could have been more fortunate in form or material, for it is a book full of interest; it instils into the mind of the people ideas of right and stimulates the best efforts toward the accomplishment of good.

---

A Manual of Hygiene and Sanitation. By Seneca Egbert, M. D., Professor of Hygiene in the Medico-Chirurgical College of Philadelphia. Third edition, enlarged and thoroughly revised. Duodecimo, 467 pages, with 86 illustrations. Lea Brothers & Co., Philadelphia and New York. 1903. (Cloth, $2.25 net.)

The interest in the subjects discussed in this modest volume is ever increasing among the laity as well as among physicians. As each class becomes better versed in hygiene and sanitation, there is more rapid progress toward the prevention of preventable diseases. It is impossible for municipalities to enforce proper sanitary regulations without the intelligent coöperation of citizens who know the importance of such measures as are enacted at the instance of the health department. For example, the ordinances relating to spitting on the streets or in the street cars are in operation in every city in which there is not a prevaling public sentiment in favor of their enforcement.

The book we are using as a text for these remarks has done much already, and will continue to do more, toward creating a wholesome public opinion on sanitary and hygienic questions. Egbert is a teacher of experience and an expert on these topics. What he writes, therefore, should carry the weight of authority. He has carefully revised this edition, adding much useful material and eliminating some that has become less so, until it now is in the forefront of books of its class. We regard it as one of the best manuals on sanitary and hygienic science in English print.

---

The Treatment of Certain Malignant Growths by Excision of the External Carotids. By Robert H. M. Dawbarn, M. D., Professor of Surgery and Surgical Anatomy in the New York Polyclinic Medical School and Hospital; Visiting Surgeon to the City Hospital, New York. (The Samuel D. Gross Prize Essay). Octavo, pages 105. Philadelphia: F. A. Davis Co. 1903. (Price, $2.00 net.)

A monograph of such exceptional quality as this one seldom falls into the reviewer's hands. It gives, in considerable detail, the labors of seven years in testing the value of excision of the external carotids, in arresting certain growths, malignant in character, and generally regarded as inoperable. Thus, the face, mouth, tongue, pharynx, and sometimes the cranial cavity, become the seat of neoplasms belonging to the class called inoperable malignant, and form appropriate fields for the operation proposed and put to practical test by the author of this monograph.

The first case in which Dawbarn put to test his operation is reported in full. It was done to arrest the growth of a roundcelled sarcoma of the nasopharynx, and consisted in reality of several operations. First, on June 1, 1895, Dawbarn tied the right external carotid at its origin. This did not have the effect of arresting nutrition, and June 10, ten days after the first, a second operation was made, which consisted of complete excision of the left external carotid artery,—the first time this operation was ever performed, as far as can be ascertained. The third operation was made January 14, 1896, which was a removal of the growth, and consisted in the entire excision of the right superior maxilla. Eight years afterward the man continued well, without evidence of return of the growth. This is a surgical achievement that should stimulate further attempts in this direction.

The author was awarded the Gross prize of the Philadelphia Academy of Surgery, of one thousand dollars, for this monograph, though when he began his investigations he had no thought of offering whatever report thereof he might subsequently make in competition for any prizes. The monograph should be in the hands of every surgeon.

---

Lea's Medical Epitome Series. Organic and Physiologic Chemistry. A Manual for Students and Practitioners. By A. McGlannan, M. D., Associate Professor of Physiologic Chemistry. Instructor in Clinical Laboratory, College of Physicians and Surgeons, Baltimore, Md. Duodecimo, pp. 246. Illustrated with 9 engravings. Series edited by V. C. Pedersen, A. M., M. D., Instructor in Surgery at the New York Polyclinic Medical School and Hospital. Lea Brothers & Co., Philadelphia and New York. 1903. (Price, $1.00.)

There two important divisions of chemistry are presented in this book in skeleton form in a most admirable manner for memorising by students who are preparing for examination. Its general plan is quite like that of similar works in the epitome series that have preceded it. It is prepared by an instructor of experience, and contains the essentials of organic and physiologic chemistry, gleaned from the best sources. For the purposes it is intended it is as complete as need be, and is unexcelled by any similar compend that we have seen.

---

Lea's Series of Medical Epitomes. Anatomy. A Manual for Students and Physicians. By Henry E. Hale, A. M., M. D., Assistant Demonstrator of Anatomy, College of Physicians and Surgeons (Columbia University), New York. Duodecimo, 384 pages, with 71 illustrations. Series edited by V. C. Pedersen, M. D., Instructor in Surgery at the New York Polyclinic Medical School and Hospital. Lea Brothers & Co., Philadelphia and New York. 1903. (Cloth, $1.00 net.)

Anatomy, the foundation of medical knowledge, must be memorised by the pupil in his earliest period of study. The tutor must instil its details by continued hammering. Every known expedient must be resorted to in order that the plastic mind may receive lasting impressions concerning even the smallest structural formation of the human body. In this book will be found a condensation of the essentials of anatomy, which will prove a great aid in acquiring the knowledge of the subject. It is one of the cleverest books of its kind with which we are familiar; its arrangement is of the best form, and every detail pertaining to perfection in bookmaking has been carried out to the letter. It is a book to be carried in the pocket and to be studied at intervals of travel between home and school, or upon journeys by rail or water. Indeed, the student should never omit the companionship of his "anatomy," whithersoever he goeth, or wheresoever he sojourneth.

---

The American Illustrated Medical Dictionary. For Practitioners and Students. A Complete Dictionary of the Terms used in Medicine, Surgery, Dentistry, Pharmacy, Chemistry, and the kindred branches, including tables of Arteries, Muscles, Nerves, Veins, Bacilli, Bacteria, Micrococci, Streptococci; Eponymic Tables of Diseases, Operations, Signs and Symptoms, Stains, Tests, Methods of Treatment, etc., etc. By W. A. Newman Dorland, A. M., M. D. Octavo, 800 pages Philadelphia, New York, London: W. B. Saunders & Company. 1903. (Price, $4.50 net; with thumb index, $5.00 net.)

It is surprising how much valuable information is compressed between the covers of this dictionary. The vocabulary alone is worth more than the price of the book, while the other material enhances its value very much. As a mere word book, it is not excelled by any dictionary of its size, and the heavy-faced type in which the words to be defined are printed makes it easy for the eye to reach the word desired with despatch. Many additions of words and tables have been made in this edition, and it is made to represent the immediate present as far as can be done by such a book. Its workmanship, too, is of the very best, its flexible cover making it pleasant to handle. It is the dictionary, *par excellence,* for the physician's library table.

Atlas of the External Diseases of the Eye. By Prof. Dr. O. Haab, of Zurich. Second edition, thoroughly revised. Edited, with additions, by G. E. de Schweinitz, A. M., M. D., Professor of Ophthalmology in the University of Pennsylvania. With 98 colored lithographic illustrations on 48 plates, and 232 pages of text. Philadelphia, New York, London: W. B. Saunders & Company. 1903. (Price, $3.00 net.)

A second edition of this admirable work proves most acceptable to every ophthalmologist, and indeed to others; for, with such graphic illustrations, the general physician is enabled to diagnosticate many diseases of the eye in their earlier stages, and so to call a specialist when he does not feel competent to conduct a given case. The descriptive text and fine chromo-lithographic plates taken together carry the student of external diseases of the eye along the pathway of the most intimate knowledge to be obtained outside the clinic itself, where all this material was collected. The second edition has been revised and a few pictures have been added, making the book as complete an atlas as can be made.

---

A System of Physiologic Therapeutics. A practical Exposition of the Methods, other than Drug-giving, useful for the Prevention of Disease and in the Treatment of the Sick. Edited by Solomon Solis Cohen, A. M., M. D., Senior Assistant Professor of Clinical Medicine in Jefferson Medical College. Philadelphia. Volume VII. Mechanotherapy and Physical Education including Massage and Exercise by John K. Mitchell, M. D., Physician to the Philadelphia Orthopedic Hospital and Infirmary for Nervous Diseases. And Physical Education by Muscular Exercise, by Luther Halsey Gulick, M. D., Director of Physical Training in the Public Schools of Greater New York. Octavo, pp. 420. With 229 illustrations. Philadelphia: P. Blakiston's Son & Co. 1904. (Price for the set, $27.50 net.)

In this admirable series many excellent volumes have appeared, but the one before us is not second in importance to any of its predecessors. It deals with methods that may be adopted for the preservation of health; or, in many conditions of invalidism, they may be invoked to cure. Exercise is too often neglected by physicians in the budget of advice given their patients; if, perchance, it is introduced, the directions are general instead of being specific, hence often the good expected is not obtained. In this book minute details are given for exercise in sickness and in health, and all the conditions are carefully set forth in a scientific fashion.

Massage is recognised as a valuable aid to the physician in many ways and under a variety of circumstances, but it often does harm when misused. It is of doubtful value, and still more questionable propriety, when applied to the female pelvic organs. Erotic women who would scorn to masturbate themselves find in osteopathy a cover for their real desires, and an ally to appease their sexual hunger. This author finds pelvic massage of little or no value, but does not condemn it on the ground above referred to.

Orthopedics are dealt with quite extensively as well as scientifically, and the illustrations are also excellent. The entire volume, indeed, abounds in good material and in fine pictures, making it not only instructive, but attractive.

---

Lea's Series of Medical Epitomes. Normal Histology. A Manual for Students and Practitioners. By John R. Wathen, M. D., Professor of Surgery and Gynecology in the Kentucky School of Medicine, Louisville. Series edited by V. C. Pedersen, M. D., Instructor in Surgery at the New York Polyclinic Medical School and Hospital. Duodecimo, pp. 229. Illustrated. New York, Philadelphia: Lea Brothers & Co. 1903. (Price, $1.00.)

This book is one that may well be found in the hands of physicians who have been some years in practice, as well as in those of the undergraduate student. Histology has not heretofore been made the subject of a compend or epitome in the manner set forth by this author. Many of the illustrations are microphotographs of actual histologic conditions, some of which are meritorious in a high degree. As an aid to the study and understanding of a complex subject it contains all that need be published in a compend. It embraces the essentials of the subject as taught at the present day, in acceptable form for those who desire an epitome for ready reference.

---

The International Medical Annual. A Yearbook of Treatment and Practitioner's Index. Thirty-two contributors, American and Foreign. Twenty-second year. Octavo, pp. 750. New York and Chicago: E. B. Treat & Co. 1903. (Price, $3.00.)

After a practical test we are convinced that this reference book compares favorably with any work of its class. It needs expert knowledge to prepare such a book, because of the immensity of the material at hand each year, and without great care the size of the volume would easily pass the proper limit. For twenty-two years this volume has presented itself and each edition has been better than the previous one, though it appears each time as if further improvement would be next to impossible. It would be well if the Annual revised its compounding of such words as *smallpox,* and omitted diphthongs in such words as *anemia;* however, these are questions that time will determine. The plates and other illustrations are useful in elaborating the text of a very excellent book.

BLAKISTON'S QUIZ COMPENDS. Compend of Gynecology. By WILLIAM H. WELLS, M. D., Chief of the Gynecological Staff of the Mount Sinai Hospital, Philadelphia. Third edition, revised and enlarged. With 145 illustrations. Philadelphia: P. Blakiston's Son & Co. 1903. (Price, 80 cents.)

Compends appear to have become a necessity during these days of work and worry, in preparation for the various examinations that cheer the pathway of the undergraduate, not to mention the post-graduate examinations for license and for commissions in the public service. If, therefore, we must have compends let us have good ones such as this. In the edition under consideration some errors have been corrected and much new material has been added, so that it may be said to represent in condensed form the best gynecological teaching of the day. The illustrations, too, are many and well chosen. It contains all that is desirable in a compend.

---

MT. SINAI HOSPITAL REPORTS. Volume III. For 1901 and 1902. Edited for the Medical Board by N. E. BRILL, M. D. New York: Stettiner Bros. 1903.

The value of hospital reports is greatly enhanced by accurate editing. This octavo volume of 522 pages is filled with interesting clinical material carefully reported and properly edited. Here and there, too, is found an illustration, but more might have been inserted without doing violence to the scientific character of the work. It would be a good plan for authors of hospital papers to give as much attention to their accurate illustration as would be given to a treatise or magazine article.

A memorial of Dr. Paul F. Mundé, who was associated with the Mount Sinai Hospital for twenty-four years, is published herein, accompanied by an excellent portrait of this distinguished physician. The book lacks a good index to make it what it should be.

---

Transactions of the Medical Association of the State of Alabama. Annual meeting held at Talladega, Ala., April 21-24, 1903. G. P. Waller, M. D., Secretary.

The state of Alabama has long been distinguished by an admirable medical association, perhaps the best organised state medical society in the union. The record before us would seem to justify this reputation. It begins with the usual secretary's minutes and other preliminary material, which take up 130 pages. In the second part, the scientific work is recorded, beginning with the annual oration by Lewis Coleman Morris, of Birmingham, the subject being, Medical and sanitary dissertations and reports. The monitor's address follows, and then the Jerome Cochran lecture, which latter was delivered by G. H. Price, of Nashville, Tenn. The first strictly medical paper, speaking clinically, is entitled, The care and management of women during pregnancy, by Edward Burton Ward, of Salem. An exhaustive essay on the American hookworm disease, by Ch. Wardell Stiles, of the United States Public Health and Marine Hospital Service, is printed in this section of the book. Neglected prophylactic and therapeutic measures forms the subject of an interesting paper by William H. Saunders, of Mobile. In the third and final part of the volume is printed the annual register of the association, which is a roll of the county societies. This splendid volume is a potent argument against state medical journals.

---

Report of the Commissioner of Education for the year 1902. Volumes I. and II. Washington: Government Printing Office. 1903.

The first volume of this report contains, in its preliminary material, statistics of the state school systems, together with a condensation of the legal provisions governing the practice of medicine and dentistry in the several political divisions. An interesting essay in the second chapter by Francis Newton Thorpe, on Franklin's influence in American education, should be read by every educator. The college-bred negro forms the subject of the third chapter, and will be read by many interested in the race problem. Chapter eleven is a translation from the German, relating to the medical inspections of schools abroad. It is an excellent essay on an important subject.

The second volume begins with a chapter on education in Porto Rico, followed by one on the same subject in Alaska. The thirty-sixth chapter deals with professional schools. Some interesting statistics appear, among which we note that the number of medical schools in 1902 was 154, with 26,821 students, a gain of 64 over 1901. The number of homeopathic students was decreased by 261, while the number in non-sectarian schools increased by 248, and in eclectic and physiomedical, 77. Although there was an increase of medical students, there was a decrease of 407 in the number of graduates, due probably to the lengthened course of study, the full effect of which has not yet been felt.

Schools for nurses form the subject-matter of chapter forty-two. The number of these schools in 1902 was 545, number of nurses receiving instruction, 13,252 and the number completing the course was 4,015. This shows an increase in schools of 100, and in pupils of 1,653.

The various subjects of interest dealt with in these volumes serve to make them unusually important, and they will be examined by all persons having relations with public and professional instruction. It is to be regretted that they must wait so long for a report of such value. The time between the period covered by the report and its printing should be shortened.

---

BOVININE. A Compend of the Practice of Hematherapy as applied to General Medicine and Surgery. Octavo, pages 574. Illustrated. New York: The Bovinine Co. 1903.

We have not observed, heretofore, on the part of the manufacturers of a proprietary preparation such a complete and detailed account of experiments, or so wide a range of application, as has been presented in this volume. It is prepared under the direction of Dr. T. J. Biggs, of Sound View Hospital, Stamford, Conn., and consists chiefly of clinical reports of cases treated by himself and others with bovinine. Dr. Biggs is employed by the company to conduct clinical and scientific work, experimental and otherwise, and will supplement this published account of his findings, by further editions of this handbook from time to time, whenever it may seem expedient or necessary.

---

TRANSACTIONS OF THE AMERICAN OTOLOGICAL SOCIETY. Thirty-sixth annual meeting held at Washington, D. C., May 12 and 13, 1903. Volume VIII., Part II. F. L. Jack, M. D., Secretary.

The work of this society is always interesting embracing, as it does, the latest and best thought on the important diseases of the ear. In the present volume, the great interest now attracting to mastoid diseases is indicated by the fact that of sixteen contributed articles six relate to diseases of the mastoid, early or late, simple or complicated, most of which required operative intervention.

The society is the oldest of the special organisations, and maintains an excellent *esprit de corps,* fifty-five members having attended this meeting,—nearly fifty per cent. of the membership. Dr. Oren D. Pomeroy, of New York, one of the charter members of the society, died during the previous year and an appropriate memorial of him prefaces the contents of the volume. An alphabetical index of otological bibliography from June 1, 1902, to June 1, 1903, compiled by B. Alexander Randall and Barton H. Potts, of Philadelphia, brings to a close this interesting record of the year's work of this splendid body of scientific workers.

---

Transactions of the American Surgical Association. Volume XXI. Edited by Richard H. Harte, M. D., Recorder of the Association. Philadelphia: Wm. J. Dornan, Printer. 1903.

The annual volume of this famous association, always replete in scientific interest, this year seems overflowing with good material. In addition to the contributions of the regular members, of which there are many of exceptional quality, there are papers by foreign visitors that add much in value to the book. The first of these is "Small contributions to the surgery of the intestinal tract," by J. von Mikulicz, Breslau, in which he details methods of dealing with four conditions: (1) cardiospasm and its treatment; (2) peptic ulcer of the jejunum; (3) operative treatment of severe forms of invagination of the intestine; (4) operation on malignant growths of the large intestine.

The second foreign paper consists of an elaborate presentation, comprising more than sixty pages, of the "Surgery of the simple diseases of the stomach," by B. G. A. Moynihan, Leeds. These two celebrated surgeons, as might be anticipated, received the distinguished attention of a large audience during the presentation of their communications. In examining this highly instructive and splendid volume, we are impressed with the scantiness of the discussions,—a remarkable fact when the gifted membership is considered.

